# Microcapillary cell extrusion deposition with picolitre dispensing resolution

**DOI:** 10.1007/s42242-022-00205-3

**Published:** 2022-09-01

**Authors:** Saeed Fathi, Iek Man Lei, Yang Cao, Yan Yan Shery Huang

**Affiliations:** 1 Department of Engineering, University of Cambridge, Cambridge, UK; 2 The Nanoscience Centre, University of Cambridge, Cambridge, UK

**Keywords:** Microcapillary cell printing, Resolution dispensing, Cell sedimentation, Single cell

## Abstract

Extrusion-based cell deposition has become a prominent technique for expanding bioprinting applications. However, the associated print resolution in the order of nanolitre or above has been a limiting factor. The demand for improving print resolution towards the scale of a single cell has driven the development of precision nozzle extrusion, although the benefits gained remain ambiguous. Here, aided by in situ imaging, we investigated the dynamics of cell organisation through an extrusion-based microcapillary tip with picolitre precision through in-air or immersion deposition. The microcapillary extrusion setup, termed ‘Picodis’, was demonstrated by generating droplets of colouring inks immersed in an immiscible medium. Next, using 3T3 fibroblast cells as an experimental model, we demonstrated the deposition of cell suspension, and pre-aggregated cell pellets. Then, the dynamic organisation of cells within the microcapillary tip was described, along with cell ejection and deposition upon exiting the tip opening. The vision-assisted approach revealed that when dispersed in a culture medium, the movements of cells were distinctive based on the flow profiles and were purely driven by laminar fluid flow within a narrow tip. The primary process limitations were cell sedimentation, aggregation and compaction, along with trapped air bubbles. The use of picolitre-level resolution microcapillary extrusion, although it provides some level of control for a small number of cells, does not necessarily offer a reliable method when a specified number of cells are required. Our study provides insights into the process limitations of high-resolution cell ink extrusion, which may be useful for optimising biofabrication processes of cell-laden constructs for biomedical research.

**Supplementary information:**

The online version contains supplementary material available at 10.1007/s42242-022-00205-3.

## Introduction

Bioprinting is an emerging technique in tissue engineering that has found broad applications from tissue regeneration to the fabrication of tissue models for drug discovery and clinical prediction [1–4]. Extrusion bioprinting, as one of the most widely used bioprinting methods [5, 6], offers a relatively versatile approach that enables the dispensing of different bioink types, including high cell density pellets, cell-laden hydrogels and spheroids [7–9]. Its ability to integrate multi-material structures opens up numerous possibilities for creating vascularised tissue models [10, 11]. Improving the deposition resolution might further present opportunities to construct cross-length scale features in the extracellular matrix and tissues [12–15] and enable the development of biosensors with micron-scale sensitivity for in situ cell sensing [16]. However, the precision of extrusion cell printing is currently limited, typically at about 150 µm resolution [17]. This technique generally involves the use of either a pneumatic or a mechanical dispenser. The latter, most commonly based on piston-driven positive displacement, provides more direct control over the flow of bioink through the nozzle for a wider viscosity range [18]. The resolution of micro-extrusion bioprinting is influenced by various factors, including printing conditions (such as nozzle geometry, nozzle diameter, dispensing flow rate, dispensing force) and material properties (such as bioink viscosity and the interfacial property of the bioink versus its medium) [17, 19]. Meanwhile, various efforts have been made to improve the mechanical precision of dispensers in microcapillary-based extrusion [20, 21], but there still remains a scope for miniaturisation in the plunger driving mechanism, as well as space for improvement in the dispensing precision and repeatability [22] for printing cells in the form of dispersed cell suspension or cell aggregates [23]. This can be further enhanced by utilising a vision-assisted approach in conjunction with micro-extrusion that allows for the characterisation and control of cell and organoid deposition in 2D and 3D structures [24, 25].

While inkjet printing can offer a higher volume dispensing resolution than mainstream extrusion-based cell printing approaches, this technique is limited to the deposition cells at a low density in a medium [26]. Hydrogel-based cell-laden extrusion is the primary approach to tackle this drawback by creating dispersed bioinks [27]; however, it also has limitations in the ability to provide high cell densities needed for the replication of tissue constructs [22]. The early adaptation of cell dense extrusion strategies included the use of spheroids [28, 29]; however, most recently, cell-pellet as a bioink has been utilised for direct extrusion without involving any carrier biomaterial [30, 31]. Cell pellet extrusion was shown to enable the creation of an aggregate that was subsequently developed into kidney organoids for drug discovery [32]. This approach of bioprinting has proved to allow for the precise manipulation of organoid size, cell number and conformation, in order to optimise the process of manufacturing uniformly patterned kidney tissue sheets with functional tubular segments. Here, aided by in situ imaging, we investigated the dynamics of cell printing using a pulled microcapillary tip through micro-extrusion with picolitre precision. The picolitre extrusion microcapillary tip, which employs a piezoelectric actuator to drive a piston in a glass microcapillary pre-filled with a deposition material, can typically dispense aqueous droplets at an extrusion resolution of about 3.6 pL per step and a rate of 1 to 2000 steps per second (Hz). In addition, our setup is coupled with an imaging system that enables the in situ monitoring of liquid flow within the microcapillary and the extruded flow from the microcapillary, providing additional insights into the challenges of precise deposition of cell suspensions and cell aggregates.

## Materials and methods

*Microcapillary tip preparation:* Glass PCR pipettes (Drummond Scientific Inc. outer diameter 1030 µm, inner diameter 500 µm) supplied with stainless-steel plunger (diameter about 485 µm) were used. Prior to coupling to the dispenser, the glass microcapillary bores were pulled (P-1000 by Sutter Instrument Corp.), creating a microscopic hair-like tip with a conical transition from the uniform cylindrical bore shape. The tips were dressed by a microforge platform (MF-900 by Narishige Group), resulting in finished lengths from 55 to 65 mm with a range of tip sizes (about 20 to 80 µm inner diameters at the tip opening (nozzle) for the studies here). For the experiments, the pulled capillary tips were back-filled with a dispensing material (i.e. test liquid or cell solution). Tygon-tubing (Tygon^®^ ND 100–80 by VWR International) with an outer diameter of 2.3 mm and inner diameter of 0.7 mm was cut to 10 mm long sections, and used to couple the unpulled section of the microcapillary tip through interference fit of 8 mm of its length from one side, with 2 mm remaining at the end of microcapillary to provide a seal for coupling with the pipettor tip or another unpulled microcapillary during the back-filling process.

*High-resolution pico-dispenser:* Based on the pulled microcapillary described above, we developed a custom piezoelectric dispenser, called ‘Picodis’ herein. The stainless-steel plunger was coupled with the Picodis piezoelectric actuator and then inserted into the rear unpulled section of the microcapillary via the Tygon-tubing section that also coupled the glass microcapillary to the dispenser housing. The Picodis utilises a piezo-flexure drive mechanism, in which each trapezoidal voltage signal triggers the ‘step’-wise piezoelectric actuation, resulting in the linear displacement of the plunger inside the straight section of the microcapillary. The rate of actuation in the Picodis can be tuned from 1 to 2000 steps/s. Thus, the total plunger linear displacement is set by the total number of input steps (via a computer interface). The plunger can move forward or backward by controlling the direction of trapezoidal signal. This allows the potential injection or aspiration of materials. It is of note that the translation of the displaced medium volume through the tip could not be determined directly by multiplying the linear displacement of the plunger by the cross-sectional area of the medium. This is due to the clearance on the plunger and microcapillary and also the compressibility of medium/air interfaces in the tip. Therefore, a calibration experiment was performed using a food colouring ink as described below.

*Microcapillary extrusion characterisation:* In order to visualise and thus characterise the dispensing resolution and control, in situ imaging (Moticam1 by Motic Europe GmbH) along with magnifying lenses (Navitar Inc, USA) was used in the platform to monitor the phenomena occurring inside and around the transparent microcapillary tip when interacting with the recipient medium. Food colouring ink (typically viscosity = 10 mPa·s [33]) was used as the dispensing solution, while the base part of polydimethylsiloxane (PDMS) kit (Sylgard 184, Dow, USA), with a viscosity of 5100 mPa·s at room temperature, was used as the recipient medium in the initial immersion printing experiments. The dispensing solution was carefully loaded into the microcapillary tip via backfilling using a typical manual pipettor. The tip was then coupled and clamped to seal with the dispenser. Any excess ink was pushed out of the tip by the steel plunger and was cleaned with soft tissue prior to immersion into the liquid bed, which was either a Petri-dish or a 96-well plate. The dispenser attached to the *XYZ* motion control platform (LNR50/M and MTS50/M-Z8, Thorlabs Ltd) was programmed in a sequence to deposit at the designated spots.

*Cells:* 3T3 fibroblast cells (ATCC) were used as the model cell line. Cells were incubated in T75 flasks at 37 °C and 5% CO_2_ (Dulbecco’s Modified Eagle’s Medium (low glucose) by Life Technologies Ltd, with 10% FBS) and then harvested typically when the cells reached 70%–80% confluency by trypsinising. Two cell dispensing conditions were used for the micro-extrusion study. The first condition was cell suspension at a typical concentration of 1.0×10^6^ to 2.0×10^6^ cells/mL in the culture media. The second cell dispersion condition was cell pellets, which were generated by centrifuging a cell suspension at 350 G for 5 min, when most of the supernatant culture media was removed. The pellet was then gently transferred into an autoclaved glass microcapillary tip via backfilling. It was estimated to have a cell density of well over 50 million cells/mL (max 250×10^6^ cells/mL given a typical 3T3 cell size).

**Fig. 1:**
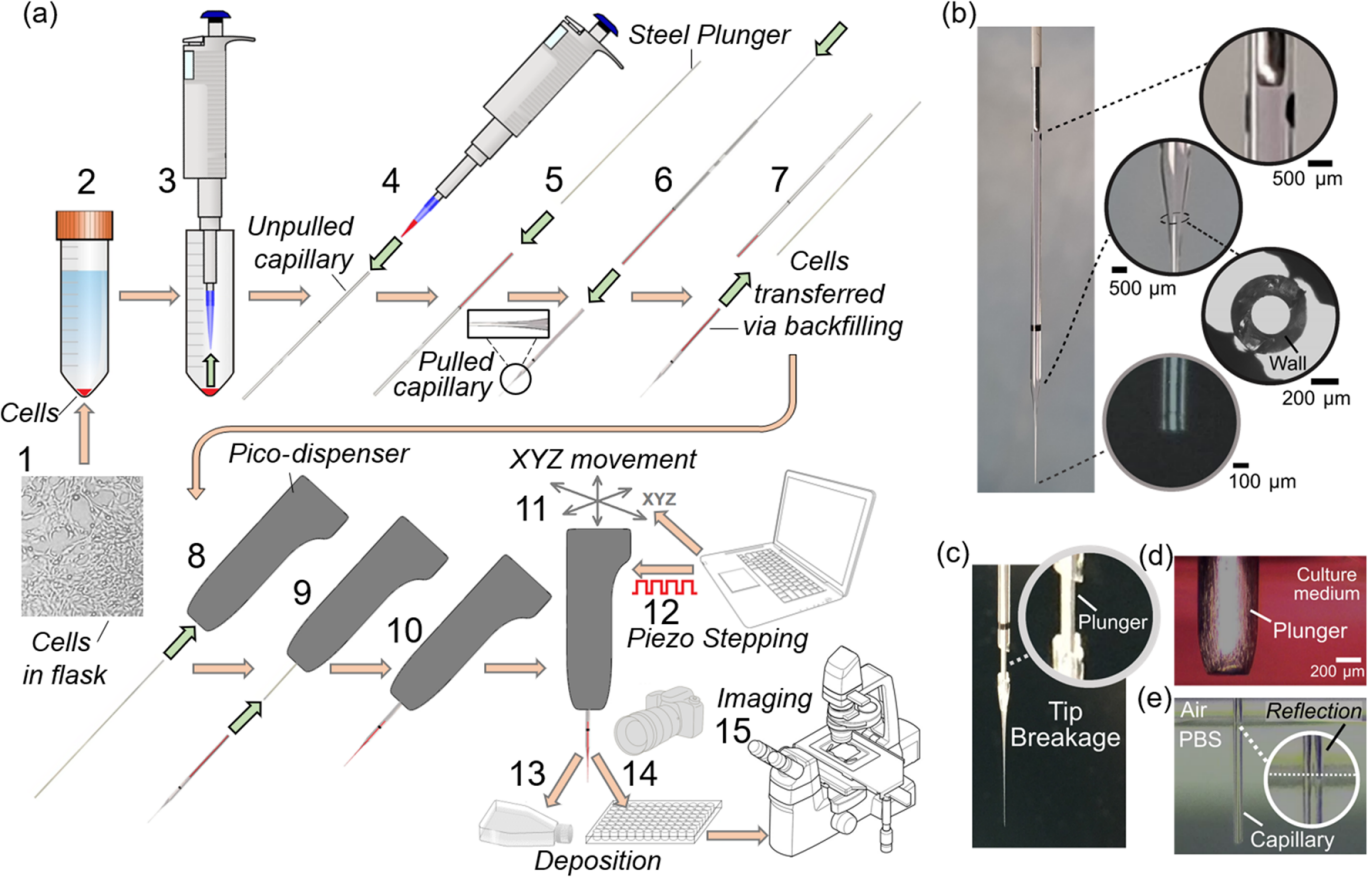
Overview of the image-assisted pico-dispenser (Picodis) setup. **a** Workflow of microcapillary dispensing to prepare a cell pellet dispensing experiment: (1) 3T3 cells in culture flask. (2) Cell centrifugation. (3) Removal of supernatant medium to yield a cell pellet that was retrieved by a pipettor. (4) Transfer of cell pellets into an unpulled glass microcapillary. (5) Insertion of steel plunger to the unpulled microcapillary. (6) Coupling of the unpulled microcapillary with the pulled microcapillary (tip), and transfer of cells from the unpulled microcapillary to the pulled capillary tip. (7) Decoupling of unpulled microcapillary and plunger, and completing the backfilling of cells into the pulled capillary tip. (8) Insertion of plunger into Picodis and securing it to the stepper actuator for linear displacement. (9) Insertion of loaded tip into Picodis. (10) Securing the tip in the Picodis housing. (11) Spatial positioning of tip into the final position. (12) Stepping control for cell extrusion. (13) Cell deposition into phosphate buffered saline (PBS) bath for cell deposition characterisation. (14) Deposition into 96-well plate vials for offline imaging. (15) Offline imaging via inverted microscope. **b** Example of a pulled glass microcapillary used in this study. **c** A broken microcapillary caused by continuous stepping after the steel plunger reached the conical part of the microcapillary. **d** A steel plunger immersed in cell culture medium. **e** A microcapillary tip immersed in PBS in a rectangular container to obtain a sharper edge definition for accurate dimensional measurement

*Cell extrusion experiments:* Fig. 1a demonstrates the workflow used for cell extrusion in this study. The transfer of cell pellets into the pulled tip involved a set of intermediate unpulled microcapillaries and a steel plunger, as demonstrated in Fig. 1a. The backfilling of each tip was performed carefully to prevent the formation of an air gap between the end of plunger and the tip bore. By inserting the tip into the dispenser, the cell medium was pushed to fill in and purge out the excess volume through the tip. The tip was consequently clamped to the dispenser. Tip openings (nozzle) with different inner diameters, ranging from 20 to 85 µm, were utilised to study how tip diameters affect the extrudability of cell medium. The deposition of compact cell pellets was performed in a 96-well plate pre-filled with culture media using 1000-step and 50-step actuations. Each cell injection experiment lasted for about 30 min, after which the dispensed cells were placed back to the incubator for further culture. Overall, the sample production involved having cells outside of the incubator typically for 1–2 h.

*Cell viability experiments:* In order to evaluate the role of piezo actuated cell extrusion on the viability of deposited cells, three sets of samples were produced in 96-well plate vials filled with 200 mL culture media buffer. A cell suspension with a concentration of 2.0×10^6^ was used to backfill the tip as described earlier. Samples in set 1 were positive control cells, which were not subjected to the actuated dispensing process and the small opening of pulled microcapillary. This set of samples was produced using unpulled microcapillary with inner diameter of 510 µm and manually depositing 1 µL by a steel plunger as positive control. The deposition of set 2 and set 3 samples was performed using 1000-step actuation via tips with 42- and 57-µm openings at 500 steps/s. For set 2, the viability of cells was determined one day after depositing them into the medium. For set 3, the viability of cells was evaluated after three days. Two-colour fluorescein assay was conducted to determine cell viability in a live/dead cytotoxicity protocol with 2 µM calcein acetoxymethyl (Invitrogen™ Calcein-AM from Fisher Scientific, USA) and 4 µM Ethidium Homodimer-1 (EthD-1 from Fisher Scientific, USA) using an inverted fluorescence microscope (Echo Revolve, USA). Live cells taking up and retaining the calcein dye in their cytoplasm resulted in bright green fluorescence, while the ethidium homodimer entered dead cells to bind to nucleic acids and produced bright red fluorescence. Counting and measuring the viability of cells were performed based on fluorescent images analysed using ImageJ software (National Institutes of Health, USA). Cells subjected to the pico-dispending process showed similar viability as the positive control group at around 90% after one day, as presented in Supplementary Information.

## Results

### Piezoelectric-driven extrusion mechanism

Figure 1b shows a typical microcapillary tip configuration prepared for the experiments. By measuring the inner diameter (ID) and the outer diameter (OD) at the conical section of the microcapillary tip (Fig. 1b), the ratio of ID to OD was found to be typically 0.48. This provided us with a calibration factor to determine the IDs of the pulled ends of the microcapillary tips in our experiments. For each experiment, the stainless-steel plunger, which was fixed to the dispenser’s drive mechanism, was inserted into the bore of the microcapillary tip. The outer surface of the microcapillary tip was then clamped to the dispenser’s housing. The clamping feature ensured an effective gripping of the tip and thus the effective transmission of displacement from the actuator. Figures 1c demonstrates that the continuous stepping of the plunger made it reach the narrower pulled section of the tip, when it broke the narrow end of the microcapillary rather than slipping its unpulled end out of the Picodis housing. This effective clamping strategy strengthened the precision of our dispenser as a positive displacement mechanism. Figures 1d and 1e show the images taken in immersion experiments where the plunger diameter was used to calibrate the tip geometry measurements. A combination of reflected light and backlight helped to obtain a sharper edge definition around the tip submerged inside a rectangular flask, as seen in Video S1 (Supplementary Information).

### Meniscus control and droplet generation

**Fig. 2:**
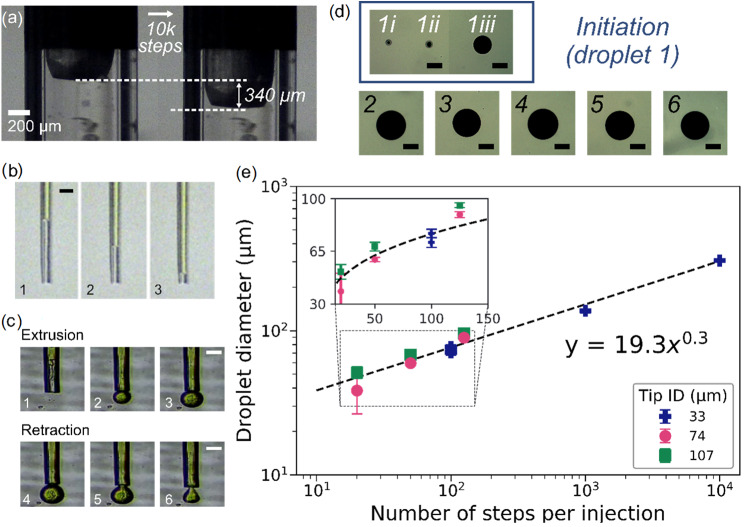
Controllability for liquid dispense. **a** In the null-loading case, an example of the plunger movement triggered by 10,000-step actuation at a rate of 500 steps/s (Video S2 in Supplementary Information). **b** In the fluid-loaded case, meniscus control with the colouring ink in the microcapillary tip (ID=39 µm) in air, with images showing two consecutive 50-step actuations, as shown in Video S3 in Supplementary Information (Scale bar=100 µm). **c** Example of the process of droplet generation and retraction using colouring ink through a microcapillary tip (ID=49 µm), as shown in Video S4 in Supplementary Information (Scale bar=100 µm). **d** Droplets generated with 1000-step actuation and lateral movement of the tip, as shown in Video S5 in Supplementary Information. Consistency was observed after first ejection (Scale bar=200 µm). **e** Correlation between the number of steps per injection and the generated droplet diameter for tips of different inner diameter (ID) openings at 1000 steps/s

The mechanical resolution of the dispenser was initially assessed by moving a plunger inside an unfilled microcapillary tip with a total actuation including 10,000 steps at a stepping frequency of 500 steps/s (Fig. 2a and Video S2 in Supplementary Information). The overall plunger displacement of 340 µm suggested that the step size of the dispenser in the ‘null-loading’ scenario was about 34 nm/step, with an average linear velocity of 17 µm/s. The translation of such control over the colouring ink as a reference liquid is shown in Fig. 2b (see also Video S3 in Supplementary Information). The facile manipulation of the meniscus position in response to a 50-step actuation represents a high level of control over the flow resolution (also analysed in Fig. S1 in Supplementary Information).

Subsequently, droplet generation experiments were performed with the colouring ink in an immersion printing setting to assess the dispensing resolution and repeatability. The food colouring ink, which is a homogenous colloidal dispersion with a quoted viscosity of 10 mPa·s [33], could act as a useful baseline for the following cell suspension and cell pellet extrusion experiments. Here, microcapillary tips were inserted into a bath of PDMS base medium, where a controlled number of steps was actuated to inject a volume of the ink. Figure 2c visualises the process of droplet formation and subsequent droplet retraction using a 49-µm tip opening and 50-step actuation procedure (Video S4 in Supplementary Information). Once a volume of droplet was ejected, its separation from the tip could be achieved using either lateral movement or vertical retraction of the tip. In the example shown in Video S5 (Supplementary Information), an array of droplets with lateral spacing of 2 mm was injected into PDMS using a 152-µm microcapillary tip, with an actuation of 1000-step per injection and at a frequency of 2000 steps/s. The resulting array of droplets is shown in Fig. 2d. Of the six injections, consistency was observed in the last five, whereas the first injection (forming two satellite droplets alongside the main droplet) resulted in the inconsistency observed. Nonetheless, after initiation, a high consistency of generated droplets was shown, as demonstrated in the 96-well plate by single droplet per vial experiments in Fig. S2 (Supplementary Information). Figure 2e summarises the effects of the number of steps per injection input and the tip opening on the droplet diameter. It is shown that, regardless of the tip diameter, the generated droplet diameter (*d*_drop_) could be related to the total number of input steps (*N*) by *d*_drop_≈19.3*N*^0.3^. This is very close to the results of theoretical correlation considering volume conservation, i.e. *Nv*_i_ = π*d*_drop_^3^/6 (*v*_*i*_ represents the volumetric resolution per step), where *d*_drop_ is directly proportional to $N^{\frac{1}{3}}$. Using the fitting in Fig. 2e, we extrapolated the mean volume resolution of *v*_i_ ~3.6 pL per step (c.f., a typical rounded cell of about 10 µm in diameter has a volume of about 0.5 pL). These initial data suggest that the Picodis system has high mechanical precision and repeatability for liquid dispensing.

### Cell suspension within a microcapillary: sedimentation, flow profile and aggregation

**Fig. 3:**
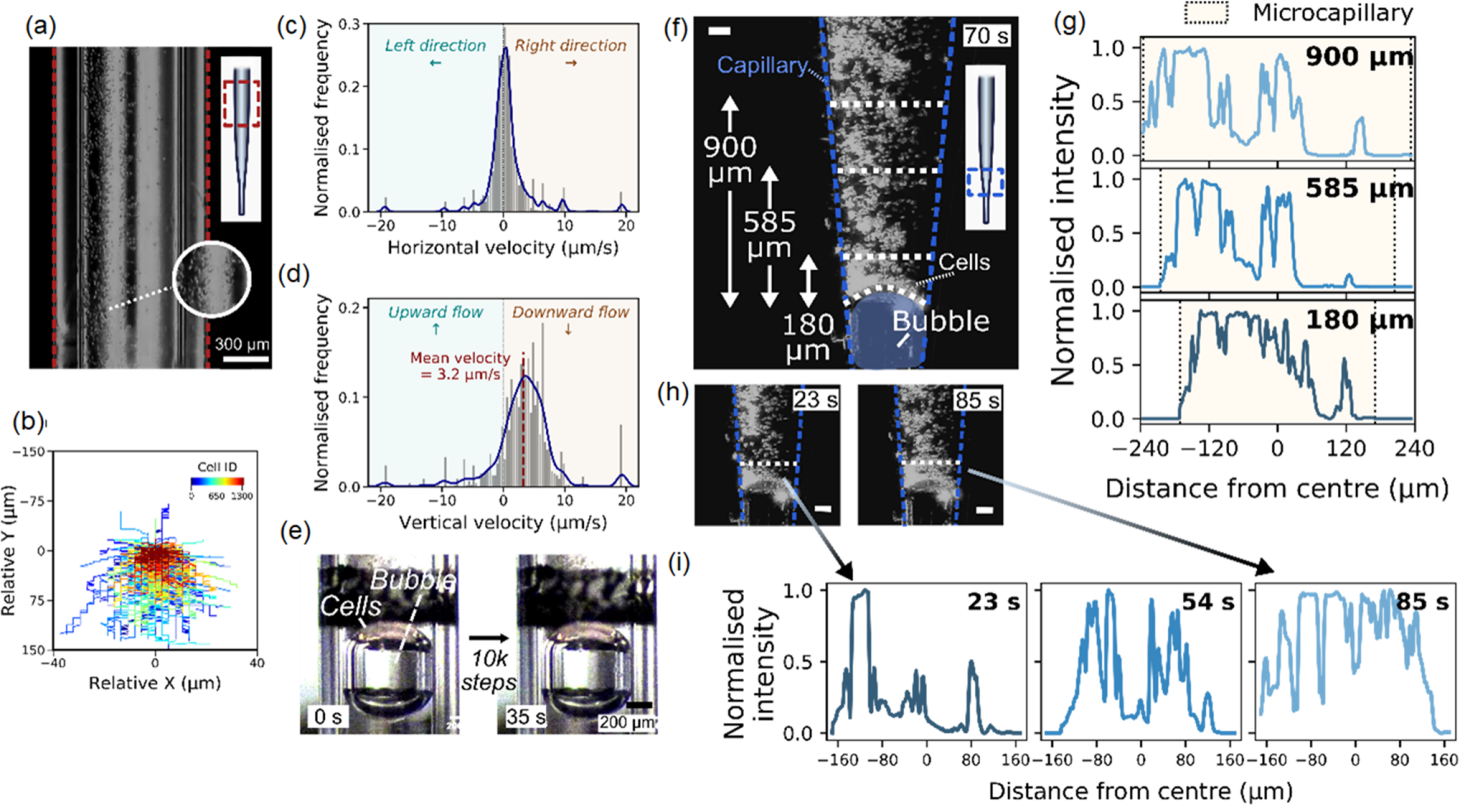
Sedimentation of cells in a microcapillary loaded with cell suspension. **a** Image showing inhomogeneous cell distribution within the microcapillary when it was left flat before fitting to the dispenser. **b** The relative positions of about 1×10^3^ cells during the sedimentation process. Normalised frequency histograms of **c** the horizontal velocity and **d** the vertical velocity of cells during sedimentation. **e** A trapped air bubble inside a microcapillary, as shown in Video S7 in Supplementary Information. **f** Close-up images showing inhomogeneous cell density behind an air bubble within a microcapillary at 70 s from the start of imaging, corresponding to Video S8 in Supplementary Information (Scale bar=50 µm). **g** Intensity profiles along the cross-sectional lines of the microcapillary at 900, 585 and 180 µm from the meniscus. **h** Images showing the time-dependent accumulation of cells above the meniscus. **i** Intensity profiles at 23, 54 and 85 s (since the start of imaging) obtained along the horizontal dashed line, which is 180 µm from the meniscus (Scale bar=50 µm)

Next, we investigated whether the precise liquid dispensing control given by Picodis can be adapted for cell-medium dispensing. Single cell control was demonstrated using a narrow tip to inject and aspire, as shown in Supporting Information. However, it was recognised that the first challenge the system should overcome is cell sedimentation in the case when a standard culture medium is used in the loaded tips without additives (e.g. surfactants) or agitation. Prior to installing the plunger for deposition, cell sedimentation could be a dominant factor governing the quality of cell deposition. When the tip was left flat on a surface prior to fitting onto the dispenser, cell sedimentation to one side of the tip was found to take place within 5 min. This was confirmed when the tip was positioned upright (attached to the dispenser), as shown in Fig. 3a. Cell sedimentation was shown to be faster on the more populated side (the automatic cell tracking results are summarised in Fig. 3b). The normalised frequency histograms of the horizontal and vertical speed of cells are shown in Figs. 3c and 3d. The distribution of horizontal speeds in Fig. 3c indicated that the cells were aggregating at an upper speed of about 10 µm/s. On the other hand, the distribution of vertical speeds in Fig. 3d revealed upward cell movement, suggesting a retrograde flow. Furthermore, the main cell population was shown to have an upper vertical speed of about 10 µm/s, which corresponds well to the theoretically estimated terminal speed (about 10 µm/s) of a single cell with 15 µm size in a creeping flow. The increase in apparent cell size, i.e. due to aggregation, could increase the terminal speed further, which could account for the observed vertical cell speed at about 20 µm/s. Video S6 (Supplementary Information) shows the situation with the cell suspension in a small microcapillary tip (24 µm), where the clustering of cells is seen at the long narrow end of the tip due to sedimentation and subsequent dispenser actuations. Repeated 250-step injection actuations eventually created a hanging drop from the microcapillary tip in air. With each actuation, the cells inside the hanging drop moved, and new cells joined as the drop became larger.

In addition to sedimentation, we found that the other common factor affecting the flow profile of cells is the presence of an air bubble within the capillary, as seen in Fig. 3e. Due to the narrow inner diameter of the microcapillary, an air bubble can be trapped within the bore of the capillary in the back-fill loading approach described in Fig. 1a. However, this effect could be hard to identify with direct visual inspection, since the flow of medium was much less hindered by the presence of air bubble. As shown in Videos S6 and S7 (Supplementary Information), although the air bubble blocked the cell passage in the microcapillary, the formed hanging drop in air continued to grow at the tip exit; thus, the culture media would have passed around the bubble. However, cells remained trapped behind the bubble to form aggregates. Figure 3f shows an experiment corresponding to Video S8 (Supplementary Information) where cell sedimentation resulted in cell accumulation on top of a trapped air bubble in the conical end of the microcapillary tip (ID=29 µm). By analysing the normalised intensity along the cross-sectional lines of the microcapillary tip at different vertical positions (indicated in Figs. 3f and 3h), we confirmed that the cell density rapidly increased towards the air bubble within tens of seconds (Figs. 3g and 3i).

**Fig. 4:**
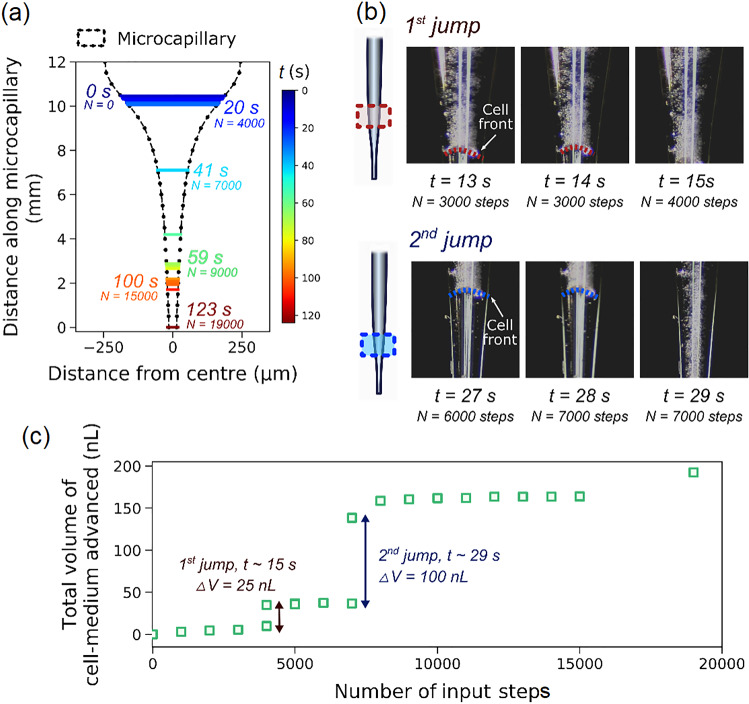
Visualisation of sedimented cell aggregation, compaction and microcapillary tip blockage during ejection experiment. **a** Inner diameter profile of the pulled microcapillary at the narrow end of the tip. The positions of cell front at different time points and the total number of input steps (*N*) were labelled. **b** Video frames captured from Video S9 in Supplementary Information showing the cell front positions during two significant jumps in the advanced cell-medium volume. **c** Plot of actual volume of cell-medium that advanced over the number of input steps applied

Figure 4 and Video S9 (Supplementary Information) demonstrate how the sedimented cells were extruded out into a hanging drop by a series of 1000-step actuations. By extracting the microcapillary profile and based on the position of the liquid–air interface over time (Figs. 4a and 4b), we obtained the actual volume of cell-medium advancing over the number of input steps applied (Fig. 4c). Here, volume was calculated from the displacement of meniscus for two data points, i.e. events captured from video, multiplied by the average of meniscus cross-sectional area at the start and the end of two data points. It was seen that the cells aggregated behind the bubble were compacted with the actuations while propagating through the narrow end of the tip (Video S9 in Supplementary Information). The actual cumulative volume of displaced cell-medium is shown in Fig. 4c, where two primary behaviours can be observed for the advancing meniscus with actuations. First, two jumps in the cell-medium volume were seen as a result of the removal of air bubbles, with a major one at about 29 s (or 7000 steps). Second, further actuations resulted in the limited displacement of meniscus towards the tip end, potentially causing the compaction of cells and further back pressure. The cell front finally reached the tip opening when the effect of stiction to the glass was eased off for a rapid release of cells through the tip, which consecutively formed a hanging drop attached to the outer of the tip in air. The ‘over-shoot’ in the cumulated volume of the cell-medium compared to that of the plunger movement, as shown in Fig. 4c, could be due to back pressure accumulated earlier as a result of microcapillary insertion into the dispenser and the presence of the large bubble.

### Cell pellet extrusion

**Fig. 5:**
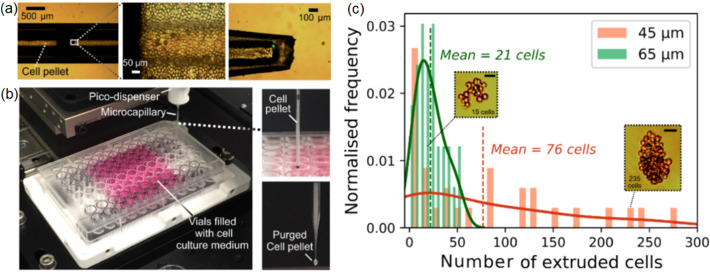
Cell pellet extrusion through microcapillary. **a** Microcapillary filled with a high density of cell aggregate. **b** Image of the experimental setup used for injecting cell aggregates into a microplate prefilled with culture media. As shown, the purged cell aggregate through the microcapillary tip in air initiated the microcapillary tip for the experiment prior to immersion into vials. **c** Variability in the number of cells ejected when different sizes of microcapillary tip were used. The number of cells was counted within 10 min after the ejection experiments. The mean values were indicated (Scale bar=50 µm)

Subsequently, we performed deposition experiments using centrifuged, pre-aggregated cell pellets, and investigated how increased cell density would affect the extrusion behaviour. Figure 5a shows a typical microcapillary tip filled with centrifuged cell aggregates. During the injection process (Fig. 5b), the tip of the microcapillary entered each vial, after which an injection of 50 steps was performed prior to retraction and movement to the next vial. Figure 5c compares the deposited cell counts in each injection for two tip sizes (as detailed in Figs. S3 and S4 in Supplementary Information), which shows a significantly higher level of cell count variation for the 45-µm tip than the 65-µm tip. In particular, for the 45-µm tip, it showed no deposited cells in the first few vials, and then cells were ejected in larger numbers at some point due to the accumulated back pressure release. Thus, for dispensing dense cell aggregates, the use of a small microcapillary tip and a reduced number of steps per injection could result in accumulating pressure in the tip.

**Fig. 6:**
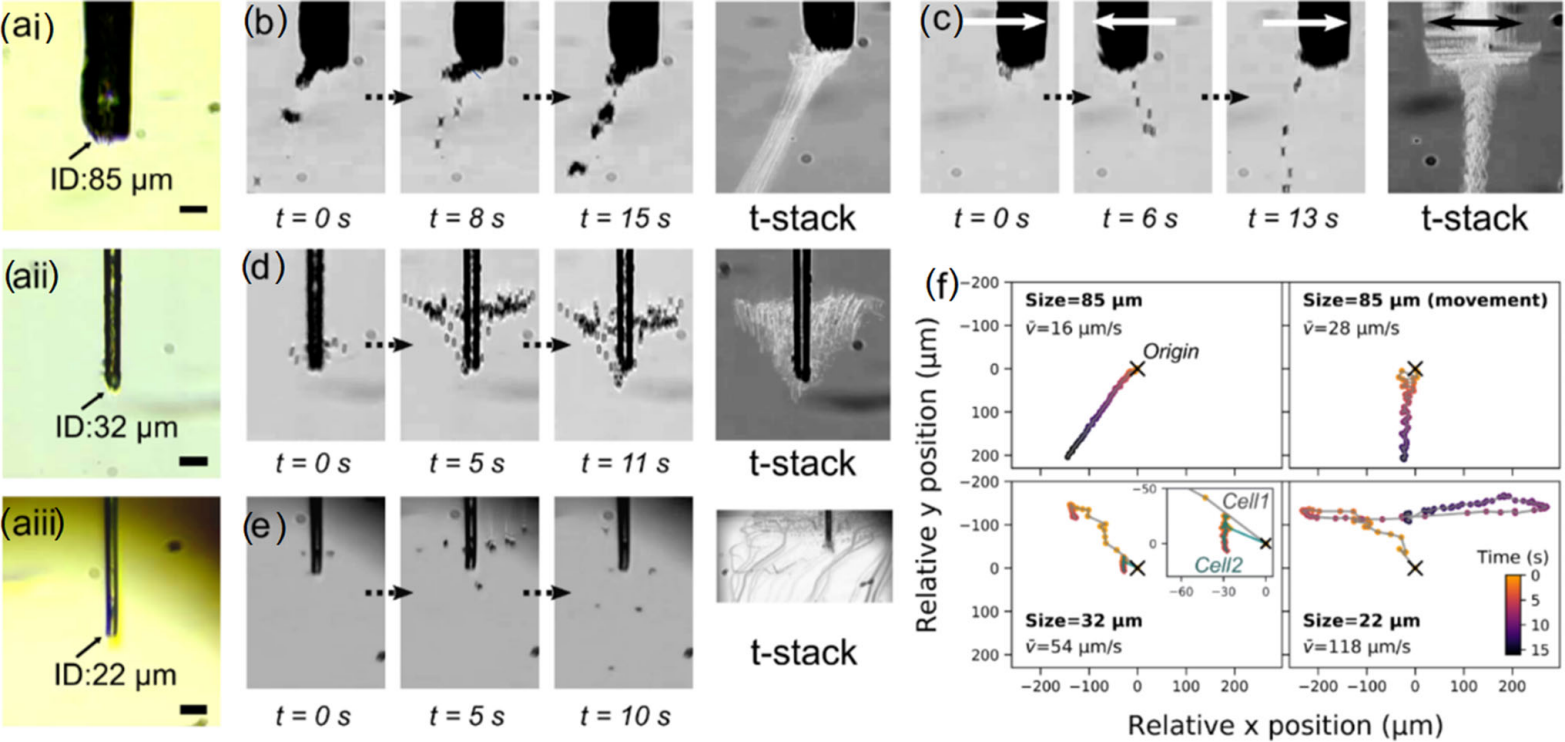
Forms of cell pellet exiting a microcapillary tip. **a** Images showing cell pellet extrusion experiments using different sizes of microcapillary tips. **b**–**e** Flow of ejected cells using **b** a 85-µm ID microcapillary tip with 100-step actuation; **c** the same parameters as **b** with 0.2 mm at 0.5-Hz side-to-side movement of the tip; **d** A 32-µm ID tip with 250-step actuation and **e** a 22-µm ID tip with 1000-step actuation. **f** Relative position of ejected cells in the b–e experiments. The mean speed of the cells, $\overline{v}$, was indicated

In order to investigate the observed deposition inconsistency, we utilised in situ imaging to probe the cell exit dynamics, where aggregates were extruded through the microcapillary tip of ID about 20–85 µm. The results are shown in Fig. 6 and Videos S10–S13 (Supplementary Information). Depending on the tip size, tip movement and the number of steps, different patterns of cell separation and flow were observed when the cell-medium exited the tip opening, as shown in Figs. 6b–6e. In general, it could be observed that the reduction in tip diameter increases the turbulency of the dispensing flow, compromising the controllability of the positional precision. As shown in Fig. 6b and Video S10 (Supplementary Information), the aggregated cells at the vicinity of the tip separated gently from the clot when a large microcapillary tip was used (ID=85 µm) along with smaller actuation steps (100-step). By incorporating a 0.2-mm side-to-side movement of the same tip without dispenser stepping, the separation of cells from the tip was seen (Fig. 6c and Video S11 in Supplementary Information). Overlapping all frames (*t*-stack as in Fig. 6c) shows the cell separation and flow pattern within an about 80-µm-wide confined region. With the tip size decreasing (to 32 µm) and the number of steps in the injection actuation increasing from 100 to 250 steps, the cells were ejected from the tip in a jet-like pattern and were immediately lifted up in the form of a vortex pattern. The width of this pattern was about 400 µm by the time the stepping stopped at *t*=462 ms (Fig. 6d and Video S12 in Supplementary Information). The sedimentation then started to settle down the cells, as seen in Video S12 (Supplementary Information). When a second 250-step actuation operation was applied about 10 s later, the originally sedimented cells were prompted to lift up along with newly ejected cells. Notably, by further reducing the tip size to about 22 µm, very few cells could exit the tip, even with an increased actuation size. Eventually, a small number of cells burst out of the tip into the medium, creating a turbulent jet flow, as seen in Fig. 6e and Video S13 (Supplementary Information).

The comparison of the three deposition patterns was quantified as shown in Fig. 6f. As inferred, the ejection of cells with larger microcapillary tips resulted in larger packs of cells and a slower mean speed of the ejected cells. On the other hand, using smaller tips, there was a faster mean speed of cell ejection, and a smaller number of steps per actuation was required to obtain a level of control. The upper speed of about 122 µm/s estimated for the cells ejecting from the smallest tip opening was still significantly slower than the speed of red blood cells (up to 20–30 mm/s [34]) in circulation in vivo. The elastic nature of cells when packed, however, makes it difficult to predict the ejection process.

## Discussion and conclusions

In summary, a new piezo-driven microcapillary tip extrusion approach, named as Picodis, was developed and studied on its ability to achieve a picolitre-level resolution of cell deposition. For Newtonian liquid dispensing, Picodis was expected to offer an excellent on-demand flow rate control, typically from 3.6 pL/s and up to 7.2 nL/s with the adjustable piezo stepping frequency (corresponding to 1 to 2000 Hz). Using pulled microcapillaries of tip sizes from about 20 to 85 µm (ID), we investigated how the dimensions of tip opening affect the extrudability of cell-loaded media and thus the dispensing resolution and repeatability. 3T3 fibroblast cells were dispensed into a PBS solution in the form of either a cell suspension or a compact cell pellet. In both cases, we observed that the controllability of the cell dispensing process was significantly compromised compared to a Newtonian liquid, as demonstrated by experiments using colouring ink. The observations around the non-Newtonian behaviours in cell deposition and the underlying reasons are further discussed below.

**Fig. 7:**
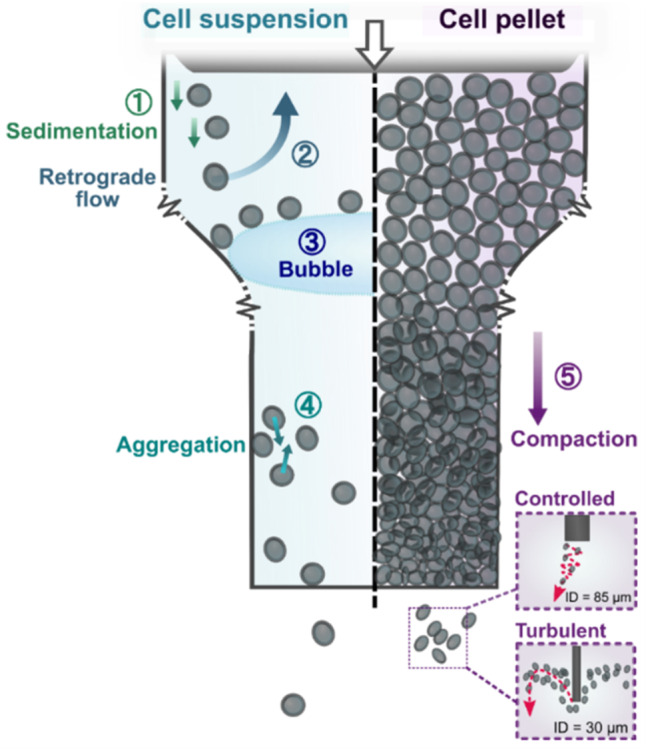
Summary of observed cell and microcapillary tip interactions during cell extrusion deposition

Figure 7 represents a summary of observations in cell extrusion deposition through a microcapillary tip. Cell sedimentation and aggregation occurred, which diminished the extrusion controllability when cells were dispersed in a non-modified culture medium (viscosity about 1 mPa·s). Air bubble, cell trapping and aggregate formation in the cell suspension extrusion remain challenges to be overcome. These could be avoided by strategies such as the use of an air trap at the back-fill loading or via acoustic approaches and inspection of the downstream vision-assisted workflow in future work. When an ultra-fine tip opening was used, the poor extrudability and turbulent extrusion flow of dense cell-loaded medium were observed, which could be attributed to the high level of compactness at the narrow end of the microcapillary. A high cell count variation was observed when using a smaller tip opening; however, this improved significantly with a larger tip opening. Taking experimentally observed aggregation and sedimentation speeds of about 10 µm/s, an initially well-dispersed cell solution of 10^6^ cells/mL (giving an average cell spacing of about 100 µm) would only yield an estimated working time of tens of seconds before the aggregation and sedimentation effects become problematic. Similar challenges were observed in the inkjet printing approach to deposit cell suspension, where a surfactant and the agitation of cells in the reservoir were used as potential workaround [26]. It was estimated that a reasonable working time (in the order of tens of minutes) could only be achieved when the cell medium viscosity increased to about 100 mPa·s. However, this could dramatically increase the shear stress experienced by the cells, potentially compromising cell viability, especially when going through a narrow tip. Although the viability tests reported in Supplementary Information revealed a fairly high level of viability after one day from deposition, there could be downstream issues with growth when depositing highly packed cell pellets through narrow tips.

Overall, in the culture medium, the cell movements are dominated by cellular dynamics including in-nozzle aggregation and sedimentation. Thus, the cells do not follow the flow paths that are purely driven by laminar fluid flow within a tip. The results reported in this study suggest that the control over mechanical resolution of a dispenser is not the dominant factor governing the extrusion resolution of cell-laden bioinks without dispersing aids such as hydrogels or surfactants. Although the Picodis setup provides a certain level of control over the cell deposition process, it needs further refinement for applications requiring more precision at the micro-scale. For example, a change in the cell loading approach could enable maximising the benefits of the high-resolution extrusion platform. Understanding the limits and characteristics of the proposed high-resolution microcapillary extrusion technique inspires future experimental designs for the microfabrication of biological constructs.

## Supplementary Information

Below is the link to the electronic supplementary material.

Supplementary file1 (DOCX 3264 kb)

Supplementary file2 (MP4 920 kb)

Supplementary file3 (MP4 610 kb)

Supplementary file4 (MP4 1056 kb)

Supplementary file5 (MP4 602 kb)

Supplementary file6 (MP4 586 kb)

Supplementary file7 (MP4 25549 kb)

Supplementary file8 (MP4 76480 kb)

Supplementary file9 (MP4 2503 kb)

Supplementary file10 (AVI 33677 kb)

Supplementary file11 (AVI 11192 kb)

Supplementary file12 (AVI 4239 kb)

Supplementary file13 (MP4 43787 kb)

Supplementary file14 (MP4 12076 kb)

Supplementary file15 (MP4 1688 kb)

Supplementary file16 (MP4 15291 kb)
