# Supplementary material for: Microcapillary cell extrusion deposition with picolitre dispensing resolution
Source: Biodes Manuf. 2022 Sep 1;6(1):1–11. doi: 10.1007/s42242-022-00205-3 (PMC9829649; doi:10.1007/s42242-022-00205-3)
Supplement: Supplementary file 1 — Supplementary file1 (DOCX 3264 kb) [file 42242_2022_205_MOESM1_ESM.docx]

**Supplementary Information**

**Microcapillary Cell Extrusion Deposition with Picolitre Dispensing Resolution**

**Note 1: Meniscus control and initiation via stepping**

Fig. S1 shows a sequence demonstrating the colouring ink meniscus control in a tip with 39 µm opening. The tip attached to the dispenser is in air and meniscus manipulation was done with forward and backward 50-step actuations. Images were captured from Supplementary Video S3 and analysed at the start (Frame 0), after 2 seconds (Frame 1), and then within 5 ms after each 50-step actuation (Frame 2 to 11) that was performed with one second intervals at a rate of 2,000 steps per second during which the meniscus did not move noticeably. Frames 1 to 5 show stepping forward representing injection while Frames 6 to 11 show stepping backward for aspiration. The figure also shows events at moments before and after one 50-step actuation (transition between Frame 2 and 3). It reveals the meniscus reaction to the 50-step actuation was longer than the actuation period of 25 ms (considering the 2,000 step per second rate). The 172 µm meniscus shift settled after about 100 ms from start of stepping. Majority of the meniscus displacement appears to have occurred during the first 50 ms from the start of meniscus displacement at 6.11 s, corresponding to a meniscus speed of around 3 mm/s in the 39 µm tip. Considering the linear stepping resolution, the meniscus displacement of 172 µm at the end of tip was initiated by only 1.7 µm of plunger displacement (considering the step size demonstrated earlier). This corresponds to an amplification factor of about 10 times due to the tip narrowing towards the end. Supplementary Video S3 shows how the high-resolution meniscus control assisted removal of air from the tip to keep the meniscus at the vicinity of the tip opening during a typical meniscus initiation and prior to extrusion.


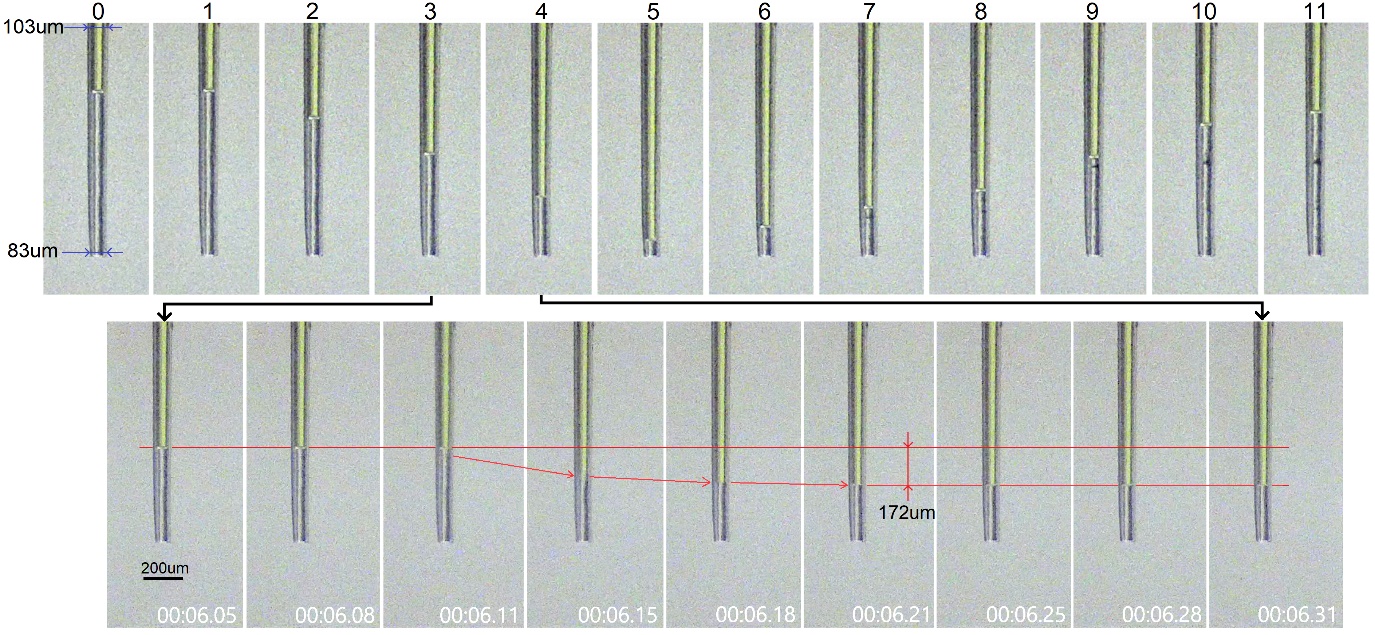


Fig. S1 Meniscus control of colouring ink inside the microcapillary tip (39 µm tip opening diameter) in air with 50-step actuations forward and backward (Supplementary Video S3)

**Note 2: Droplet Generation Consistency**

To assess droplet size consistency with small tip opening, droplets were generated in a 96-wellplate filled with 0.3 ml of the PDMS base medium, as shown in Fig S2, where the experiment involved immersing the colouring ink loaded tip with 33 µm tip opening into each vial, followed by a single injection with 100-step actuation, and finally retracting the tip upward, out of the vial to move to the next vial. Vial G8 shows two adjacent droplets, one was created in an earlier experiment. The graph representing the droplet size consistency across the vials, shows the average diameter of the dispersed droplets was around 70 µm.


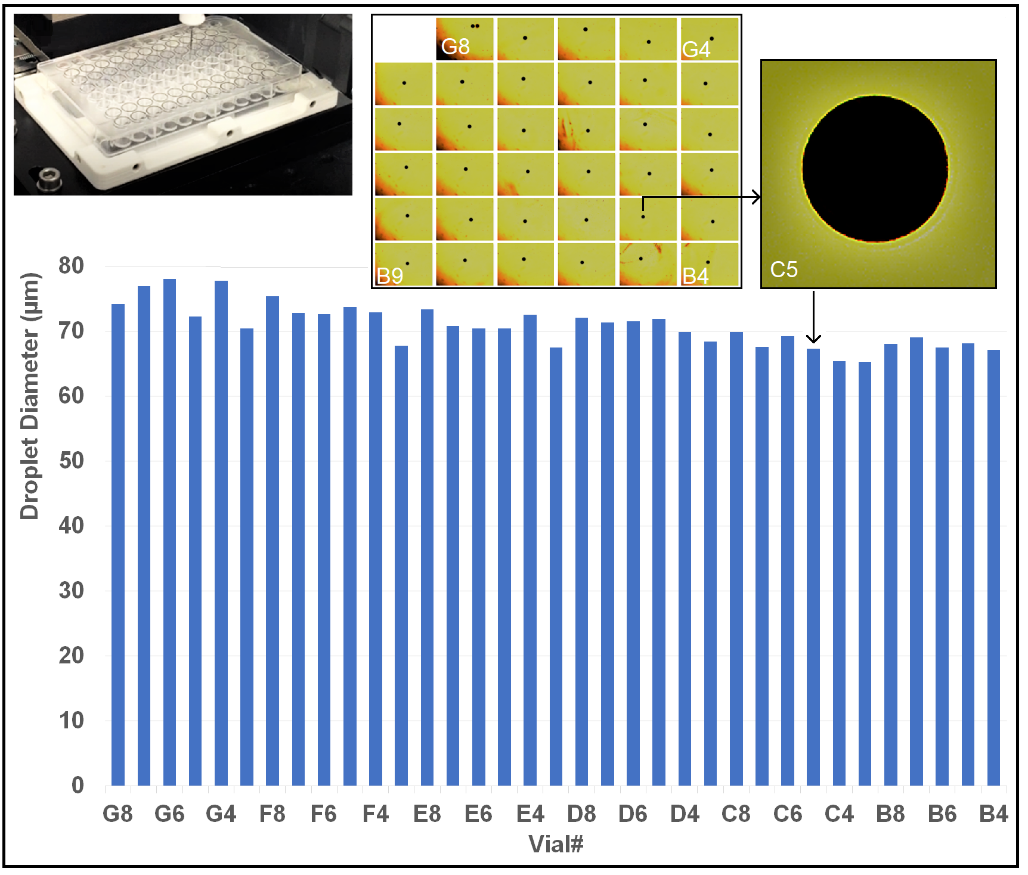


Fig. S2 Droplet generation assessment in wellplate vials filled with 0.3ml PDMS base medium, (h) droplet generated in each vial using a 33 µm tip and 100-step injections, (i) droplet diameter consistency

**Note 3: Cell deposition and viability assessment**

Precision of Picodis system control on individual 3T3 cell was tested and demonstrated in Fig. S3. Supplementary Video SI1 shows multiple cells along the tip’s end leading to nozzle is shown to be controlled across the tip by forward and backward stepping of the steel plunger. Upon ejection of a single cell, the system was able to capture the cell within a volume around the tip’s nozzle as demonstrated in Supplementary Video SI2.


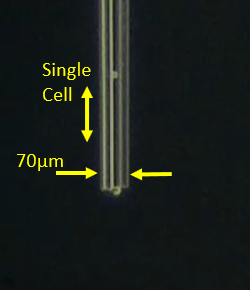


Fig. S3 Single cell control (injection and aspiration) in a tip with 33µm nozzle in an experiment with cell suspension.

Qualitative assessment of the deposition consistency is shown in Fig. S4 due to relatively large deposits with 1000-step injection. Deposited cells from the 50-step injections were counted as per Fig. S5.


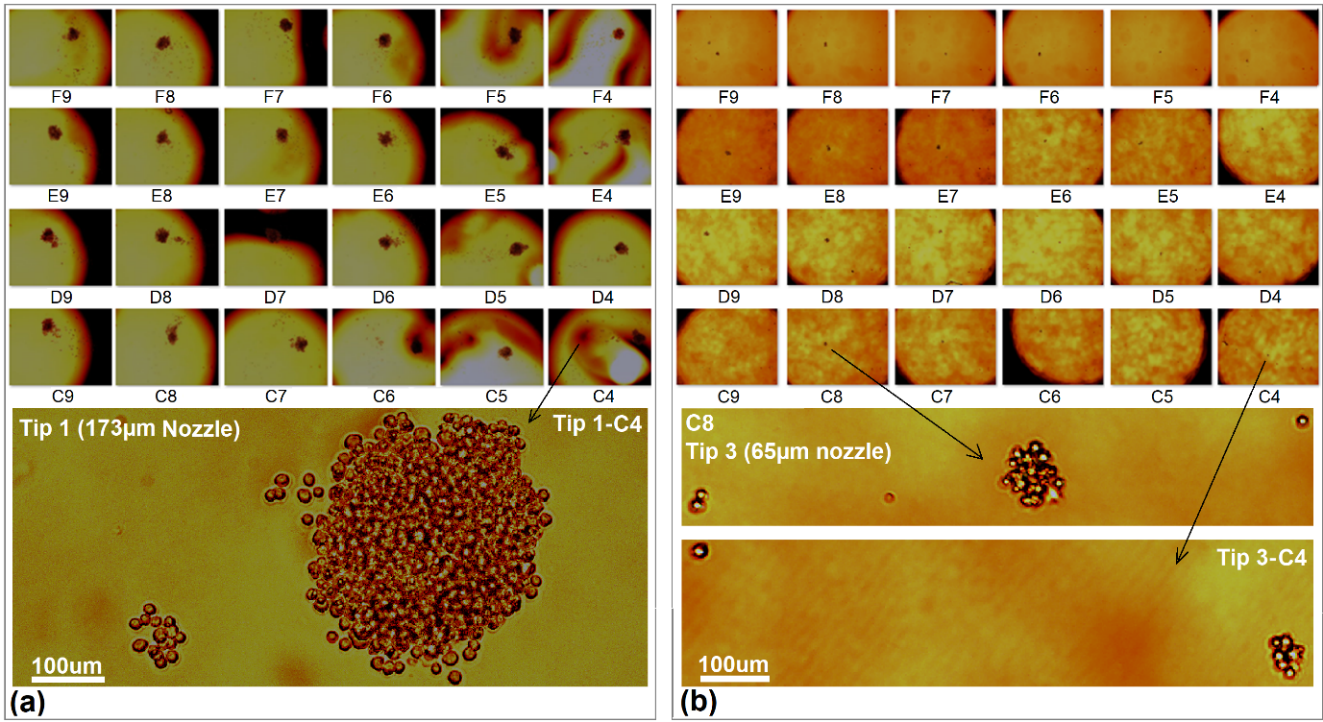


Fig. S4 Cell aggregate deposition into wellplate vials at two different deposition conditions, (a) using 173 µm tip opening and 1000-step injections, (b) using 65 µm tip opening and 50-step injections.


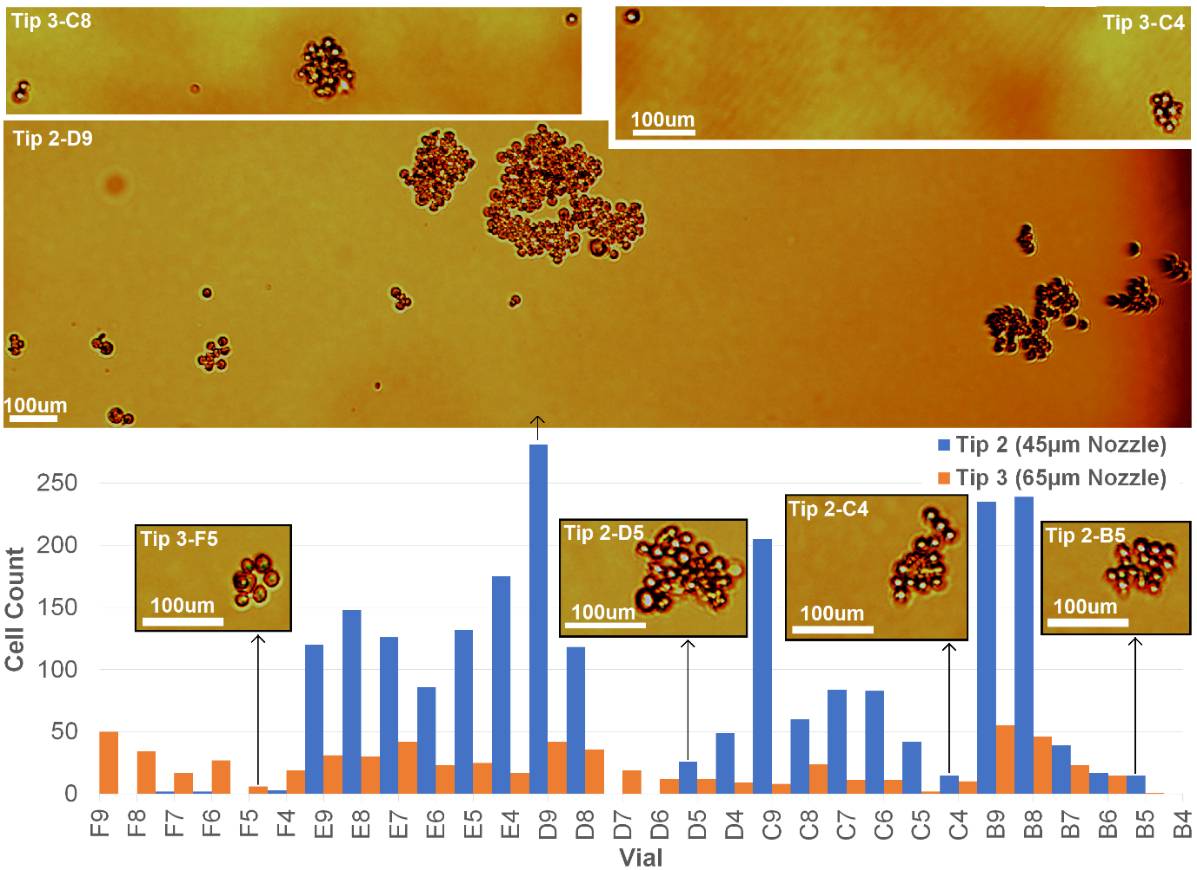


Fig. S5 Cell count variability within experiments in cell aggregate deposition using small tips with 50-step injections into wellplate vials (compare with tip 1 (173 µm) by 1000-step injections as shown in Figure S3.

**Note 4: Cell viability assessment**

To evaluate the viability of cells suspension that sedimented in the microcapillary tip in form of pellet, deposits of 1000-step were produced in various cell medium vials and compared against manually deposited cells from the suspension. **Error! Reference source not found.** presents the viability assay results and images of deposited cells into the vials in day 1 and day 3. Cells which were not subjected to the pico-dispending process, as positive control group, has shown to have similar viability of about 90% as the cells deposited via 42 and 57 µm microcapillary tip using pico-dispensing after one day. Viability of pico-dispended cells was found to be around 50% in Day 3.


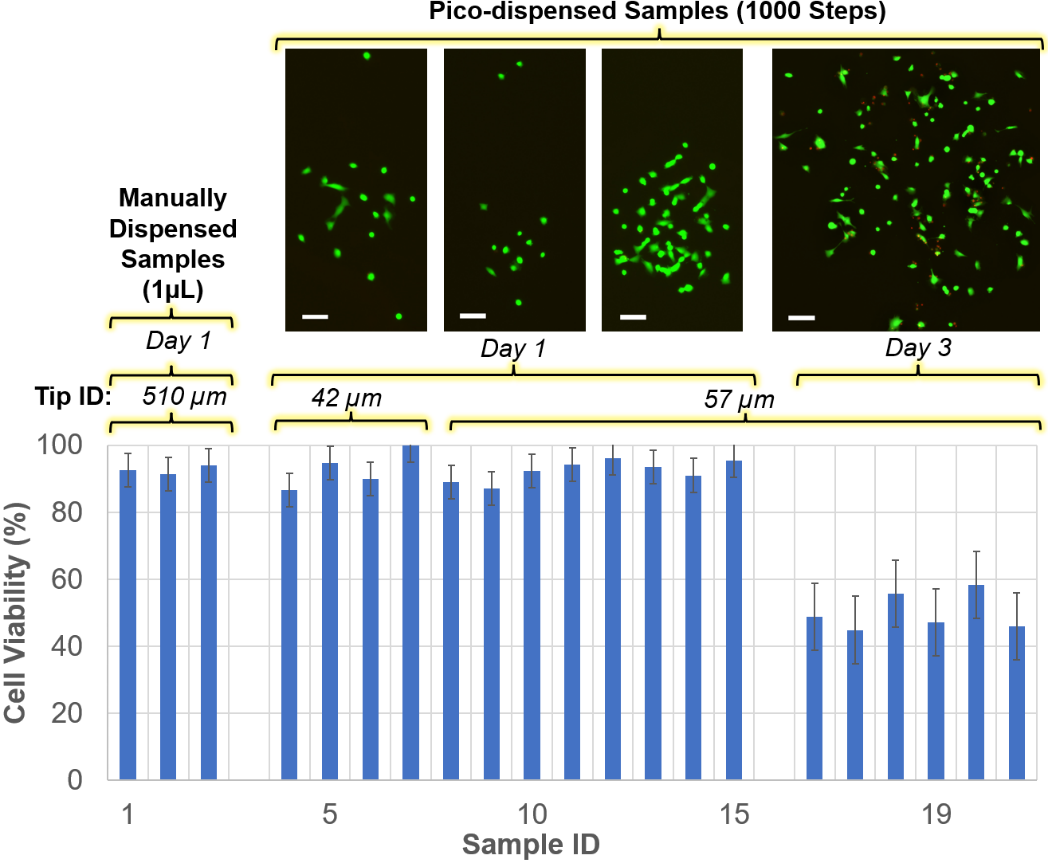


Fig. S6 Viability assessment of 3T3 cells. Images of pico-dispensed cell deposits using 1000-step actuations are typically shown (scalebar: 100µm).
